# Evaluating the Quality of Online Information on Percutaneous Endoscopic Lumbar Discectomy (PELD) Using the DISCERN Instrument

**DOI:** 10.7759/cureus.87045

**Published:** 2025-06-30

**Authors:** Alessio Tarantino, Rodrigo Buharaja, Alessandro Aureli, Attilio Basile, Ignazio Tornatore

**Affiliations:** 1 Orthopedics and Traumatology, Policlinico Casilino, Rome, ITA

**Keywords:** discern, health communication, health information quality, patient education, peld, spine surgery

## Abstract

Introduction: Percutaneous endoscopic lumbar discectomy (PELD) is a minimally invasive surgical procedure increasingly referenced in patient-oriented websites. However, the quality of such online health information is inconsistent. This study evaluates the quality of Italian-language web content on PELD using the DISCERN tool.

Methods: A systematic search was conducted on 25 May 2025 using the Italian-language interface of Google (Google LLC, Mountain View, California, United States) with the keywords "PELD," "discectomia endoscopica percutanea," and "ernia del disco PELD." Twenty websites targeted at the general public were selected. Each was independently assessed using the 16-item DISCERN instrument by two trained reviewers. Inter-rater reliability was calculated using Cohen’s kappa coefficient.

Results: The mean overall DISCERN score across the 20 websites was 39.5 (SD = 8.7) out of 80, indicating moderate-to-low quality. High-scoring domains included clarity of procedural explanation (mean = 4.2/5), while critical aspects such as citation of sources (mean = 2.1/5) and discussion of uncertainties (mean = 1.7/5) were underrepresented. Cohen’s kappa was 0.82, indicating strong agreement between reviewers.

Conclusion: Online Italian-language resources on PELD exhibit moderate quality and frequently omit essential components such as treatment risks and alternative options. Enhancing transparency and evidence citation is essential to improve the quality of patient education materials.

## Introduction

The internet has become a vital source of health-related information, with millions of patients turning to online platforms to learn about symptoms, diagnoses, and treatment options. Among various surgical treatments for spinal disorders, percutaneous endoscopic lumbar discectomy (PELD) has gained popularity for its minimally invasive approach and clinical effectiveness [1,2]. Nowadays, it has reached a wide audience, with an increasing number of surgeons utilizing this technique, with an increasing number of procedures performed [3]. However, the rapid dissemination of information online often comes at the cost of accuracy, transparency, and evidence-based content.

Patients seeking information on PELD may encounter websites with commercial agendas, outdated data, or incomplete discussions. In this context, evaluating the quality of available content is essential to protect patients and support shared decision-making. In Italy, any public advertising of health products, such as over-the-counter (OTC) or non-prescription medicines and medical devices, must receive prior approval from the Ministry of Health, with strict rules on claims, disclosures, and even social media content (including TikTok, ByteDance Ltd., Beijing, China); this legal provision is often overlooked in practice, with many websites and social media platforms promoting health products and procedures without proper authorization or compliance with the established regulations. This study applies the DISCERN instrument [4], a standardized tool developed for assessing consumer health information already used in a wide number of other surgical techniques or key topics of recent orthopedics [5,6], to analyze the quality of online Italian-language content on PELD, aiming to scientifically and reliably assess the quality of information on this specific topic.

## Materials and methods

The study was conducted in Policlinico Casilino Hospital, Rome, Italy. A systematic search was performed on 25 May 2025 using the Google (Google LLC, Mountain View, California, United States) search engine (Italian-language interface) with the keywords “PELD,” “discectomia endoscopica percutanea,” and “ernia del disco PELD.” The first 20 relevant websites meeting the following inclusion/exclusion criteria (Table 1) were selected.

**Table 1:** Inclusion and exclusion criteria PELD: percutaneous endoscopic lumbar discectomy

Inclusion Criteria	Exclusion Criteria
• Italian language	• Academic-only content
• Dedicated content about PELD	• Access behind paywalls
• Targeted at patients or the general public	• Irrelevant or non-functional pages
• Freely accessible	

We obtained the following 20 results after performing a critical review of the data obtained (Table 2).

**Table 2:** Query results

N.	Site Name	Web Adress
1	Andrea Bolognini – Neurochirurgo	andreabolognini.it
2	NSA Neurochirurgia	nsaneurochirurgia.it
3	Casa di Cura La Madonnina – Gruppo San Donato	lamadonnina.grupposandonato.it
4	Centro Medico Giancotti	centromedicogiancotti.it
5	BSP Brain Spine Peripheral	bspneurochirurgia.it
6	Clinica Parioli	clinicaparioli.it
7	Casa di Cura Careggi – Firenze	lanazione.it
8	Casa di Cura Villa Donatello	villadonatello.com
9	Dott. Georgios Bakaloudis – Neurochirurgo	georgiosbakaloudis.it
10	Casa di Cura Quisisana – Ferrara	quisisanafe.com
11	Dott. Vito Fiorenza – Neurochirurgo	vitofiorenza.com
12	Regione Toscana – Scheda HTA Tessys	regione.toscana.it
13	Korian Sanità – Trattamento ernie discali	sanita.korian.it
14	Dr. Raffaele Scrofani – Neurochirurgia	neurochirurgiaitalia.it
15	Casa di Cura San Francesco	clinicasanfrancesco.it
16	Ospedale San Raffaele – Milano	hsr.it
17	Policlinico Campus Bio-Medico – Roma	unicampus.it
18	Istituto Clinico Humanitas – Rozzano	humanitas.it
19	Ospedale Galeazzi – Milano	ospedalegaleazzi.it
20	Karl Storz – Discectomia Endoscopica	karlstorz.com

Evaluation tool

The DISCERN instrument [4] contains 16 questions divided into sections: Section 1 (questions 1-8): reliability; Section 2 (questions 9-15): quality of treatment information; Section 3 (question 16): overall quality rating. Each item was rated from one (low quality) to five (high quality). Two independent reviewers performed the assessments. Discrepancies were resolved by discussion.

**Figure 1:**
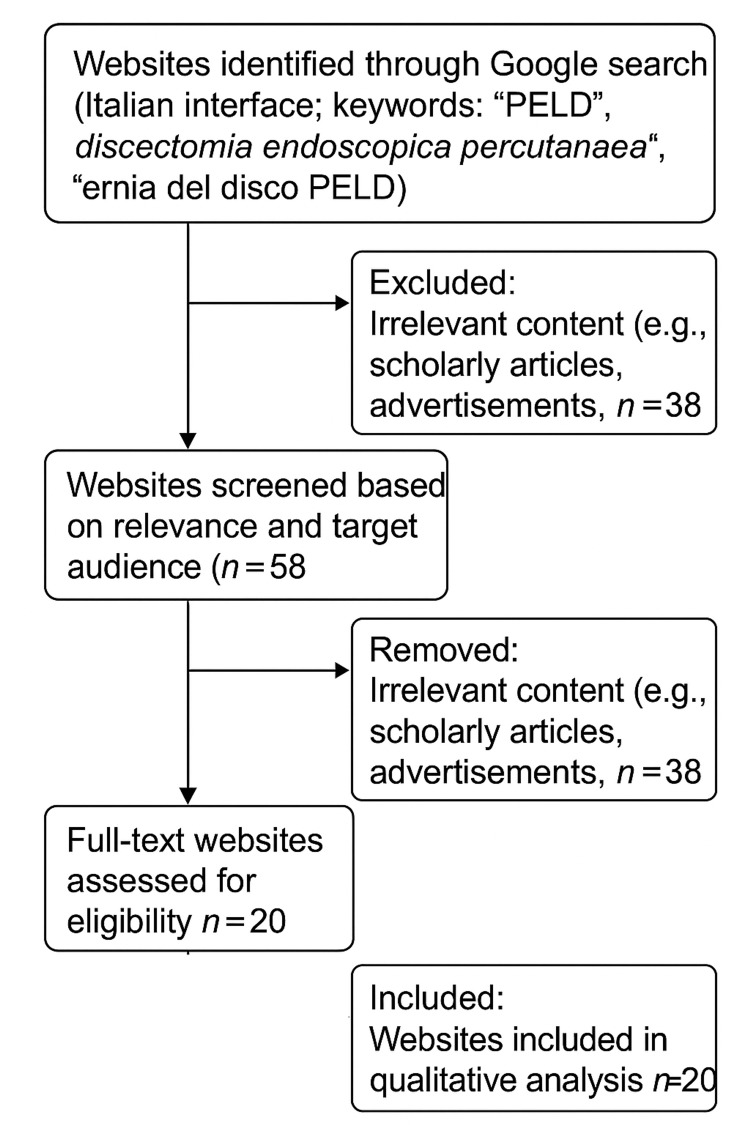
Data analysis flowchart ernia del disco PELD (Italian): herniated disc (English); discectomia endoscopica percutanea (Italian): percutaneous endoscopic lumbar discectomy (PELD, English)

A data analysis flow diagram summarizing the selection process is included in Figure 1, in accordance with the latest Preferred Reporting Items for Systematic reviews and Meta-Analyses (PRISMA) guidelines.

Evaluation and scoring

Websites were evaluated using the DISCERN tool, which includes 16 items across domains: reliability (Q1-Q8), treatment quality (Q9-Q15), and overall quality (Q16), each rated from one to five (Appendix 1). Two independent reviewers scored all websites. Inter-rater reliability was assessed using Cohen’s kappa.

Data analysis

Descriptive statistics were reported as means and standard deviations. No inferential statistics were applied.

## Results

An assessment of 20 websites providing information on PELD using the DISCERN tool revealed significant variability in the quality of the content. The majority of these websites scored in the "poor" to "fair" range (16/20), indicating shortcomings in areas such as clarity, comprehensiveness, and reliability. Only a few websites achieved "good" (3/20) or "excellent" (1/20) ratings, demonstrating well-structured, balanced, and evidence-based information. Common deficiencies across the evaluated websites included a lack of detailed explanations of treatment risks and benefits, insufficient citation of information sources, and limited discussion of alternative treatment options. Sixteen out of 20 websites showed a complete lack of bibliographic references to support their claims, which represents the main source of bias among the sites analyzed. These findings underscore the need for improved quality in online health information regarding PELD to better support patient decision-making. 

The mean total DISCERN score for all evaluated websites was 39.5 (SD = 8.7) out of 80. The domain-wise mean scores and standard deviations are presented in Tables 3, 4.

**Table 3:** Reliability domain (questions 1–8) Subtotal mean (SD): 2.9 (0.8)

Question	Mean Score (/5)	Observation
Are the objectives clearly stated?	4.2	The majority stated intent clearly.
Does the content fulfill its intended objectives?	4.0	Content matched stated goals.
Is the content appropriate and on-topic?	3.5	Some included marketing material.
Are references to sources cited?	2.1	Few referenced scientific literature.
Is the date of production clear?	2.4	Most lacked update timestamps.
Is the information presented in a fair and balanced manner?	3.0	Many emphasized benefits, downplayed risks.
Are further sources provided?	2.0	Few external links or references.
Are uncertainties discussed?	1.7	Most omitted areas of controversy.

**Table 4:** Treatment information domain (questions 9–15) Subtotal mean (SD): 2.9 (0.7)

Question	Mean Score (/5)	Observation
Is the treatment mechanism clearly described?	4.2	Clearly explained, often with diagrams.
Are the advantages of the treatment clearly outlined?	4.5	All emphasized reduced recovery time.
Are potential risks or side effects adequately addressed?	2.8	Inconsistent or lacking in detail.
Is the likely course without treatment explained?	1.6	Rarely discussed.
Does the content address effects on daily functioning or well-being?	2.9	Limited discussion.
Are other treatment options discussed?	2.3	Few mentioned conservative or open surgery.
Is there support or guidance for shared decisions?	2.0	Limited encouragement to consult physicians.

The overall quality of the DISCERN evaluation was bad, with a mean score of 2.5 (SD = 0.8), while Cohen’s kappa coefficient was 0.82, reflecting strong inter-rater agreement across all DISCERN items and confirming the overall low quality of the information reported on the websites. The data obtained did not show significant differences based on the type of website, the medical affiliation (individual surgeon, clinic, or hospital), the number of procedures reported, or the geographical location of the institution referenced by the site. No evident discrepancies were observed in the overall picture, which appeared uniformly characterized by mediocre or unsatisfactory results. 

## Discussion

This analysis reveals substantial variability in the quality of Italian-language websites on PELD. While procedural descriptions were usually clear and consistent, most sources failed to address treatment alternatives, risks, or evidence limitations.

Our findings are aligned with prior assessments of online surgical content, such as studies on robotic joint replacement websites (Tarantino et al., 2023) and stem cell therapies (Venosa et al., 2021), both of which noted a trend toward overemphasizing benefits while omitting balanced discussions [5,6].

Studies in other specialties have similarly identified a lack of shared decision-making support and insufficient citation of scientific sources. For example, Charnock et al. (1999) and Pan et al. (2020) highlight the importance of structured, evidence-based communication for safe patient engagement in decision-making [2,4]. A study published in the Journal of Turkish Spinal Surgery [7] analyzed the 50 most-viewed videos on YouTube (Google LLC, Mountain View, California, United States) related to PELD. The videos were evaluated using the DISCERN and JAMA scoring systems to assess the quality of the medical information presented; the mean DISCERN score was 30.2 out of 75, indicating an overall low quality of information; 38% of the videos were rated as "very poor," 44% as "poor," 16% as "fair," and only 2% as "good." It was observed that videos with audio achieved significantly higher DISCERN scores compared to those without audio. However, no significant differences in DISCERN scores were observed based on the source of upload, whether from physicians, healthcare institutions, or other users. These findings suggest that the majority of YouTube videos related to PELD provide low-quality medical information, underscoring the need for more reliable and evidence-based online educational resources for patients. Data obtained for this study are broadly superimposable to our data. Thus, patients relying solely on web-based information may develop misconceptions about the safety, efficacy, or candidacy criteria for PELD. In the absence of counterbalancing perspectives, such materials may lead to unrealistic expectations or misinformed choices, considering that obtaining information about medical techniques and technologies is becoming increasingly common among patients nowadays [8]. Clinicians should actively address patient-acquired misinformation and advocate for institutional quality standards in digital patient education. Website authors must strive for transparency, update content regularly, and reference peer-reviewed sources. Numerous studies have highlighted that medical information available online is often unreliable or of insufficient quality for patients. A systematic review published in PubMed [9] examined 153 cross-sectional studies evaluating a total of 11,785 websites using 14 different quality assessment tools, including the DISCERN instrument. The results showed that no website achieved an "excellent" quality rating, and only 18% were certified with the Health On the Net (HON) code. The quality of information varied according to the type of website affiliation, with government websites demonstrating higher quality compared to academic or media-affiliated sources. Moreover, the quality differed across medical specialties, with internal medicine and anesthesiology websites generally providing higher-quality information. It must also be observed that the perception of the quality of the information can change due to different patients' attitudes and features. A cross-sectional review of the literature [10] found that individuals most likely to seek health-related information online were women, younger adults, those with higher levels of education, and individuals who were employed, while different categories of patients, based on ethnicity, gender, and educational level, may have completely different approaches in terms of trust and compliance with information obtained from the internet [11]. However, low levels of health or digital literacy can lead to misinterpretation of medical data found on the internet [12]. The findings emphasize the need for targeted educational programs and validated assessment tools to enhance users’ competencies and promote a more critical approach to evaluating online health content.

Limitations

This study presents some limitations that should be acknowledged. Firstly, the number of websites analyzed was relatively small. This is partly due to the highly specialized nature of the technique under consideration, PELD, which inherently limits the availability of online content dedicated to it. As a niche surgical procedure, PELD is not widely discussed outside of professional or academic contexts, making it challenging to identify a broader range of publicly accessible sources suitable for evaluation. Nevertheless, we believe that the quality of communication regarding this technique can still be thoroughly assessed even with a limited sample. The focused nature of the topic allows for a more in-depth qualitative analysis, and the scarcity of sources itself highlights an important aspect of how specialized medical information is disseminated online. Therefore, while the restricted number of websites may constrain the generalizability of the findings, it does not undermine the relevance or value of the insights derived from this study.

## Conclusions

The majority of Italian-language websites that provide information on PELD tend to offer content of only moderate or suboptimal quality. These websites frequently fail to include essential elements such as proper citations of medical sources, detailed explanations of alternative treatment options, and transparent discussions of the limitations or uncertainties associated with the procedure. As an increasing number of patients rely on the internet as a primary source of medical information, the importance of ensuring that online health content is accurate, balanced, and comprehensive becomes ever more critical. It is therefore imperative that healthcare professionals, medical institutions, content creators, and web developers work together to raise the overall quality and reliability of online patient education materials. This collaborative effort should focus on promoting the use of evidence-based information, clear and accessible language, and respect for patient autonomy and informed decision-making. Only through such coordinated initiatives can we ensure that digital health resources truly serve the needs of patients, supporting rather than undermining their understanding of complex medical procedures like PELD.

## Appendices

Appendix 1

The DISCERN tool is organized into two main sections that evaluate different aspects of the quality of health information. The first section, focused on reliability, includes questions that assess whether the aims of the publication are clearly stated and if those aims are successfully achieved. It also examines the relevance of the content, the transparency of sources used, and the timeliness of the information. Additional criteria in this section consider whether the material is balanced and unbiased, whether it offers further resources for support, and if it acknowledges areas where uncertainty exists. The second section evaluates the quality of information specifically related to treatment options. This includes whether the publication explains how each treatment works, outlines their benefits and risks, and discusses the consequences of opting out of treatment. It also assesses whether the potential impact on quality of life is considered, whether multiple treatment options are acknowledged, and if the information supports a shared decision-making process between patients and healthcare providers.
